# Moral Disengagement and Risk Prototypes in the Context of Adolescent Cyberbullying: Findings From Two Countries

**DOI:** 10.3389/fpsyg.2019.01823

**Published:** 2019-08-08

**Authors:** Lambros Lazuras, Antonella Brighi, Vassilis Barkoukis, Annalisa Guarini, Haralambos Tsorbatzoudis, Maria Luisa Genta

**Affiliations:** ^1^Department of Psychology, Sociology and Politics, Sheffield Hallam University, Sheffield, United Kingdom; ^2^Faculty of Education, Free University of Bozen-Bolzano, Bolzano, Italy; ^3^Department of Physical Education and Sport Science, Aristotle University of Thessaloniki, Thessaloniki, Greece; ^4^Department of Psychology, University of Bologna, Bologna, Italy

**Keywords:** cyberbullying, moral disengagement, prototype/willingness model, adolescents, willingness

## Abstract

Cyberbullying is associated with a wide range of mental health difficulties and behavioral problems in adolescents and research is needed to better understand psychological correlates of this behavior. The present study used a novel model that incorporated Social Cognitive Theory and the prototype/willingness model to identify the correlates of behavioral willingness to engage in cyberbullying in two countries. Adolescent students were randomly selected from secondary schools in Italy (*n* = 1710) and Greece (*n* = 355), and completed anonymous measures of moral disengagement, descriptive norms, risk prototype evaluations and behavioral willingness to engage in cyberbullying. Hierarchical linear regression analyses showed that willingness to engage in cyberbullying was associated with moral disengagement, prototype evaluations and descriptive social norms in Italy, and with gender, moral disengagement and descriptive social norms in Greece. Regression-based multiple mediation modeling further showed that the association between moral disengagement and cyberbullying willingness was mediated by prototype evaluations in Italy and by descriptive norms in Greece. The implications of our findings are discussed in the context of self-regulating cyberbullying perpetration in adolescents and informing school-based policies and interventions to prevent cyberbullying behavior.

## Introduction

Cyberbullying is defined as the voluntary use of information and communication technology and social media to virtually attack a person or a group of people, and shares common features with traditional, face-to-face bullying, such as intentionality and goal-directedness (i.e., intending to harm the victim; Campbell, 2005; Li, 2007; Juvonen and Gross, 2008; Pyzalski, 2011; Slonje et al., 2013). Cyberbullying also shares common features with indirect bullying, which is typically based on rumor spreading, social exclusion, and denigration of the victim, and does not require physical proximity between the victim and the perpetrator (Smith et al., 2002; Smith, 2004; Slonje and Smith, 2008). Cyberbullying, however, has also some unique features that distinguish it from traditional bullying experiences, such as exposure to a potentially infinite audience, and the difficulty to discern the perpetrator’s identity (Shariff, 2005; Patchin and Hinduja, 2006; Li, 2007; Tokunaga, 2010). The available evidence suggests that cyberbullying can have detrimental effects on the mental health and well-being of the victims, including low self-esteem, poor academic performance, depression, social isolation and withdrawal, and suicide ideation and attempts (Beran and Li, 2007; Hinduja and Patchin, 2008, 2010; Smith et al., 2008; Brighi et al., 2012).

The first reported studies on the topic appeared within the last decade and focused largely on prevalence estimates and trends, impact of cyberbullying on victims, gender differences, and on identifying the different types and forms of cyberbullying (Ybarra and Mitchell, 2004; Strom and Strom, 2006; Li, 2008; Slonje and Smith, 2008; Smith et al., 2008; Menesini and Spiel, 2012; Schenk and Fremouw, 2012). Other studies examined the psychosocial constructs associated with cyberbullying and paid attention to moral values and moral disengagement (Pornari and Wood, 2010; Menesini et al., 2011), empathy (Schultze-Krumbholz and Scheithauer, 2009; Ang and Goh, 2010; Steffgen et al., 2011), self-control (Vazsonyi et al., 2012), personality (Corcoran et al., 2012), and normative beliefs and attitudes (Huang and Chou, 2010). However, there is a paucity of research on theory-driven process-models of cyberbullying that can explain how the different correlates of this behavior are meaningfully linked together and reflect common processes across contexts, cultures and populations.

### Moral Disengagement and Cyberbullying

Bandura’s (1991) social cognitive theory provides a framework for understanding perceived moral agency and the self-regulation of thought and action. Bandura (1999, 2002, 2004) also described psychological processes that explain the self-regulation of affect, cognition and action in the context of socio-moral transgressions. In this respect, moral disengagement represents a core mechanism by which transgressors can re-evaluate and cognitively re-construct their transgressions in order to alleviate negative emotional responses, such as guilt, and protect their sense of self-integrity (Bandura et al., 2000; Bandura, 2006, 2016). Moral disengagement is reflected in three groups of inter-related psychological processes which pertain to cognitively and affectively re-constructing the transgression (e.g., morally justifying the transgression or comparing it to more harmful conducts), underestimating the effects of the transgression on victims (e.g., distorting the consequences of the conduct or displacing responsibility), and denigrating the victim/target of the transgression (e.g., blaming the victim; Bandura et al., 1996; McAlister et al., 2006). Previous research in adolescent populations has shown that moral disengagement is positively associated with aggression in children and adolescents, including face-to-face bullying (for a meta-analysis, see Gini et al., 2014). Runions and Bak (2015) further argued that online environments can create the conditions under which moral disengagement enables cyberbullying perpetration. In support of this argument, different studies have shown that moral disengagement was associated with both intentions and actual cyberbullying perpetration in adolescents (Pornari and Wood, 2010; Lazuras et al., 2013; Robson and Witenberg, 2013; Kowalski et al., 2014; Bussey et al., 2015), and school-based interventions against cyberbullying were effective in changing moral disengagement processes, such as distorting the consequences and misattributing the blame for cyberbullying incidents (Barkoukis et al., 2016). Nevertheless, other studies found that moral disengagement was positively associated with face-to-face bullying but not with cyberbullying (Perren and Gutzwiller-Helfenfinger, 2012), thus, warranting further empirical investigation of the role of moral disengagement in online aggression/cyberbullying.

### Risk Prototypes and Willingness to Engage in Cyberbullying

Although moral disengagement and cyberbullying behavior seem to be correlated, research has shown that this relationship is indirect and that the effects of moral disengagement on adolescents’ intentions to engage in cyberbullying are mediated by more proximal and behavior-specific beliefs, such as social norms and prototype perceptions of the people who typically engage in cyberbullying (Lazuras et al., 2013). The prototype/willingness model (PWM) was firstly introduced in late 1990s and attempted to explain the initiation of adolescent health risk tendencies, such as smoking and unsafe sex (Gibbons et al., 1995, 1998). One of the main contributions of the PWM is that it provides an alternative to the Theory of Planned Behavior (TPB; Ajzen, 1991), which has been widely used to explain different types of health behaviors and risk-taking in adolescents and younger adults (Montano and Kasprzyk, 2015). Whereas the TPB focuses on intentionality and premeditation of the potential costs and benefits of a given action as the driving forces of decision-making and action initiation, the PWM introduces the concept of “social reaction” which explains how and why young people may engage in risk behaviors without necessarily having previously formed any relevant intentions or goal plans (Gerrard et al., 2008). To this end, the PWM recognizes that adolescent risk behavior may be elicited in response to situational cues to action and, for this reason, considers the construct of behavioral willingness as a more appropriate indicator of adolescent risk-taking tendencies than measures of behavioral intentions (Gibbons et al., 1998, 2009). Behavioral willingness represents the inclination to undertake risks under specific, risk-conducive circumstances (e.g., when being with friends who perform this behavior), and is assumed to be influenced by normative influences, such as the behavior of peers (descriptive social norms), and by risk prototype evaluations (e.g., the stereotypical view of a person engaging in the behavior in question; Gibbons and Gerrard, 1995; Gibbons et al., 2004, 2009). In the context of cyberbullying, the PWM would predict that a young person, who favorably evaluates cyberbullies and perceives these actor-prototypes as psychologically similar to him/herself, would display greater behavioral willingness to engage in cyberbullying given the chance (e.g., while in the company of peers who engage in cyberbullying). However, so far there has been limited research on the relationship of PWM constructs in adolescent cyberbullying. It is important to note that whereas the TPB emphasizes the role of subjective (or injunctive) social norms that reflect perceived social approval/disapproval of a given behavior by referent others, the PWM utilizes descriptive social norms as a source of normative influence on behavior. Descriptive social norms represent the perceived prevalence or popularity of a given behavior in referent groups, and they can explain why people may reactively and automatically (i.e., without necessarily requiring intentionality) engage in certain behaviors by simply following the lead of referent others (Rivis and Sheeran, 2003; Cialdini, 2007). Descriptive social norms have been found effective in predicting several health-related behaviors such as adolescents’ fruit and vegetables’ consumption (Lally et al., 2011; Stok et al., 2014), adolescents’ risky sexual online behavior (Baumgartner et al., 2011), alcohol consumption (Brooks-Russell et al., 2014) and physical activity (Priebe and Spink, 2011). Overall, as Cialdini (2007) and Schultz et al. (2007) pointed out social norms guide behavior as people use perceptions of what other people approve and do as a criterion for their own behavior, and descriptive social norms, in particular, are an important construct influencing human behavior.

### The Present Study

In the present study we empirically examined an integrated theoretical model that incorporated moral disengagement and PWM dimensions. This integration stems from previous research which showed that the PWM constructs can be effectively integrated in other relevant theoretical approaches and increase the predicted variance in behavior (Thornton et al., 2002; Rivis et al., 2006). Furthermore, such integration can further extent theoretical models in a specific domain, and help in distinguishing between distal and proximal influences on behavior (Gibbons et al., 2004; Buunk and Gibbons, 2007). To this end, Lazuras et al. (2013) integrated constructs from the theory of planned behavior and the PWM with moral disengagement, from Bandura’s (1991) Social Cognitive Theory, to predict cyberbullying intentions among Greek adolescents. They showed that the effects of moral disengagement on intentions to engage in cyberbullying were mediated by prototype similarity but not evaluations (i.e., how favorably/unfavorably cyberbullying perpetrators were evaluated). Nevertheless, the study by Lazuras et al. (2013) used intentions as a dependent/criterion variable and this approach has certain limitations because cyberbullying is a socially undesirable behavior and, therefore, intentions can be underreported. As previously explained, behavioral willingness refers to a more reactive response to a behavior as compared to intentions that reflects a deliberate reaction. It is expected that people may report higher scores on willingness as compared to intentions and, thus, it may be a more appropriate predictor of adolescent’s transgressive behavior, such as cyberbullying. The findings by Lazuras et al. (2013) are in line with research showing that prototype similarity is more predictive of behavioral intentions, whereas prototype favorability is more predictive of willingness (for a meta-analysis see Todd et al., 2016). In the present study, we further extended the model presented by Lazuras et al. (2013) by specifically examining the direct and indirect, via descriptive social norms and prototype evaluations (i.e., prototype favorability), association of moral disengagement with Greek and Italian adolescents’ willingness to engage in cyberbullying. In accordance with the recommendations of Todd et al. (2016), in the present study we only included prototype favorability as a predictor of willingness. It was hypothesized that moral disengagement will predict adolescents’ willingness to engage in cyberbullying (Hypothesis 1), and that this association would be mediated by descriptive social norms and prototype favorability (Hypothesis 2). These hypotheses were tested in two different countries, Greece and Italy, with the aim to examine whether the proposed process model can be replicated in different contexts. Research on cyberbullying has demonstrated significant differences among countries on the prevalence of cyberbullying (Sorrentino et al., 2019). However, empirical evidence on the psychological processes underlying cyberbullying manifestation across different countries is rather scarce. For instance, Shapka et al. (2018) examined adolescents’ motivation to cyberbully in different countries and Barlett et al. (2014) attitudes toward cyberbullying and self-construal. Although mean differences appeared, there is a dearth of research on the psychological processes underlying cyberbullying. In this line, Bauman and Bellmore (2015) and Lee and Shin (2017) suggested that more research is needed to better comprehend the nature of cyberbullying in different countries.

The present study consists of a preliminary test and an exploration of these hypothesized relationships in these countries. Greece and Italy were selected as they share similar educational systems, population structure, and socio-economic and demographic backgrounds (Fournier et al., 2018; Sorrentino et al., 2019)^1^. Furthermore, both countries have a high prevalence of cyberbullying behavior as compared to other European countries (Del Rey et al., 2015). Despite these similarities, they are still two different countries representing different student mentalities. Therefore, this approach allows us to test these hypotheses to different samples and offers stronger support for the generalizability of the model to different student populations.

## Materials and Methods

### Participants

The sample of the study consisted of adolescent and young students attending public secondary schools in Italy and Greece. The age range of the participants was between 14 and 20 years (*M* = 14.7, *SD* = 1.20; 55.5% females). A random stratified selection procedure was employed. In the first step the region of the schools was selected. In the second step the school and in the last step the students were selected. With respect to the Greek sample (*n* = 355, *M* = 14.76 years, *SD* = 1.20, age range 15–18 years, 55.5% females), students were recruited from schools in Athens and Thessaloniki, the two largest cities in Greece (totaling approx. 70% of the Greek population); providing thus a representative sample of the Greek student population. Five hundred students were approached and 355 accepted to participate in the study and completed the full questionnaire (71% response rate). The Italian sample (*n* = 1710, *M* = 16.35 years old, *SD* = 1.49, age range 14–20 years, 54.5% females) was recruited from 39 secondary schools in two central regions of Italy (Emilia-Romagna and Tuscany). Schools included all type of secondary education (lower secondary and upper secondary schools, such as lyceums, technical institutes and vocational schools) and they were located in different socio-economic areas. In both countries data collection took place at the same period (academic season 2010–2011).

### Measures

Participants completed a paper and pencil questionnaire including measures of moral disengagement, descriptive norms, prototype favorability and willingness toward cyberbullying.

#### Moral Disengagement

Moral disengagement was assessed with the 24-item respective measurement by Bandura et al. (1996), which reflected six mechanisms of moral disengagement: moral justification (e.g., “*It is alright to fight to protect your friends*”), advantageous comparison (e.g., “*Stealing some money is not too serious compared to those who steal a lot of money*”), displacement of responsibility (e.g., “*If kids are living under bad conditions they cannot be blamed for behaving aggressively*”), diffusion of responsibility (e.g., “*A kid in a gang should not be blamed for the trouble the gang causes*”), distorting consequences (e.g., “*It is okay to tell small lies because they don’t really do any harm*”), and attribution of blame (e.g., “*If kids fight and misbehave in school it is their teacher’s fault*”). In Greece, a translated version used in previous research (e.g., Lazuras et al., 2013) was employed. Responses in the Greek scale were scored on a continuous 3-point scale (from 1 = *disagree* to 3 = *agree*), and higher scores reflected higher levels of moral disengagement. In Italy, a 5-point Likert scale (from 1 = *strongly disagree* to 5 = *strongly agree*) was used to record responses and higher scores reflected higher levels of moral disengagement. In both countries the same instrument was used to test moral disengagement; however, we maintained the scoring system used during the previous test of the scale in each country. Based on the recommendations by Bandura et al. (1996), an overall sum score of moral disengagement was computed in each country. The internal consistency reliability for the 24-item version was acceptable (Cronbach’s α = 0.71 for the Greek sample and α = 0.85 for the Italian sample).

#### Descriptive Social Norms

Descriptive social norms were assessed with three distinct items reflecting informational influence on cyberbullying. Specifically, the first item (classmate norms) asked participants to estimate how many of their classmates engage in cyberbullying behavior (responses ranged from 1 = *nobody* to 5 = *almost all of them*); the second item assessed cyberbullying behavior among the five closest friends (“close friend norms,” responses ranged from 0 = *nobody*, to 5 = *all five of them*); the third item assessed perceived prevalence of cyberbullying among same-age peers (perceived prevalence norms) in Greece/Italy (i.e., “*Out of 100%, how many people your age in Greece/Italy do you think engage or have engaged in cyberbullying?*” responses given in an open-ended format); and the fourth item asked participants whether they had witnessed or heard of other same-aged peers engaging in cyberbullying (“peer norms,” responses ranged from 1 = *never to* 5 = *very often*). A composite score of the three items was used in the analyses. Due to the different response options in these items, they were transformed to *z*-scores, and these values were used to produce the construct’s mean score.

#### Risk Prototype Favorability

Following the recommendations by Gibbons et al. (1998), a definition of prototypes was given, and students were asked to evaluate the prototype of a typical same-age adolescent who engages in cyberbullying. Risk prototype favorability reflected positive or negative evaluations that were, respectively, assessed with 12 items reflecting both positive (e.g., smart, popular, cool, and independent) and negative attributes (e.g., confused, careless, immature, and dull). Responses were rated along a continuous 7-point scale (1 = *not at all*, 7 = *very much*). A mean score was calculated, and internal consistency scores were adequate for both positive (Cronbach’s α = 0.66 for the Greek sample and α = 0.66 for the Italian sample) and negative prototype attributes (Cronbach’s α = 0.61 for the Greek sample and α = 0.68 for the Italian sample).

#### Willingness Toward Cyberbullying

Students willingness to participate in cyberbullying incidents was measured with three scenarios describing situations which could trigger such behaviors (“*Suppose you have had a bad fight with a friend in school. How likely is it that you will send that person nasty messages by internet or mobile phone when you get home*?,” “*Suppose you receive a threatening or insulting text by internet or mobile phone. How likely is it that you would get even by sending a similar text to the sender*?” and “*Suppose your friends were thinking to send a threatening text or upload an insulting video or photo on the internet about a person you all dislike. How likely is it that you agree with this idea and help them*?”). Responses were anchored on a 7-point Likert scale ranging from 1 (*not at all likely*) to 7 (*very likely*). A composite score was computed with higher scores indicating higher willingness to participate in cyberbullying incidents (Cronbach’s α = 0.61 for the Greek sample and α = 0.60 for the Italian sample).

### Procedure

Ethical approval and permission to conduct the study was granted from the respective committee of the Greek Ministry of Education. After the selection of schools, the principals were informed that their schools had been selected to take part in a large scale European funded project and permission was requested. After obtaining principals’ permission, the selection of the students was made. Students were informed about the purpose of the study and informed consent was obtained. Also, parental consent was requested; students delivered to their parents a letter explaining the purpose and the procedure of the study with a note to be returned signed to the researchers in case parents did not want their child to participate in the study. No signed forms were returned. The completion of the questionnaire lasted approximately 20 min. The procedure was supervised by trained personnel alongside with the students’ teachers. Both written and oral instructions were given to students regarding the completion of the questionnaire. Students were encouraged to ask any clarifying questions and were reassured about the confidentiality of their responses which would be used solely for research purposes.

### Data Analysis

Descriptive statistics of the study’s variables were tested with SPSS 22. Internal consistency of all scales was tested using Cronbach’s alpha coefficients. Pearson’s *r* correlations were used to assess the associations between moral disengagement, descriptive norms, risk prototype favorability and willingness to engage in cyberbullying, followed by a bootstrapped (5000 resamples) hierarchical linear regression analysis of the hypothesized relationships between the constructs. Bootstrapping is a robust alternative to standard parametric estimates, when the assumptions around the latter may be violated (Fox, 2008). Regression-based multiple mediation analysis (Preacher and Hayes, 2008) was further used to assess the mediating effect of PWM constructs (descriptive social norms and risk prototype favorability) on the association between moral disengagement and willingness to engage in cyberbullying. All data were analyzed in SPSS 22 (IBM Corp., Armonk, NT, United States). MPlus 8.1 software was used to assess the measurement invariance of the descriptive social norms, risk prototype favorability and willingness toward cyberbullying.

## Results

### Measurement Invariance and Inter-Correlations Among the Study Variables

The measurement invariance of the descriptive social norms, risk prototype favorability and willingness toward cyberbullying was tested across the two samples. The measurement invariance of each measure was tested independently of the others. For descriptive social norms the results of the multiple group analysis demonstrated that the scales were invariant across the two samples (χ2 (4) = 14.34, *p* = 0.0063, CFI = 0.985, RMSEA = 0.050, SRMR = 0.035). Similar findings were reported for willingness toward cyberbullying (χ2 (4) = 41.22, *p* = 0.0000, CFI = 0.957, RMSEA = 0.095, SRMR = 0.034), whereas measurement invariance was not supported for risk prototype favorability. Means and standard deviations and intercorrelations among the study’s variables are presented in Table 1.

**TABLE 1:** Means, standard deviations and correlation coefficients among the study’s variables.

	**1**	**2**	**3**	**4**	**5**	**6**	**7**	***M***	***SD***
1. Age	–	0.12^∗^	0.00	−0.15^∗^	–0.17^∗∗^	0.06	–0.01	14.76	1.20
2. Gender	–0.00	–	0.24^∗∗∗^	–0.17^∗∗^	–0.16^∗∗^	0.26^∗∗∗^	–0.07	–	–
3. Willingness	–0.01	–0.12^∗∗∗^	–	–0.15^∗∗^	–0.22^∗∗∗^	0.21^∗∗∗^	–0.00^∗∗∗^	4.19	1.99
4. Moral disengagement	–0.10^∗∗∗^	–0.24^∗∗∗^	0.35^∗∗∗^	–	0.27^∗∗∗^	–0.28^∗∗∗^	0.22^∗∗∗^	1.86	0.25
5. Positive Prototype Evaluation	–0.08^∗∗^	–0.08^∗∗^	0.18^∗∗∗^	0.22^∗∗∗^	–	–0.35^∗∗∗^	0.24^∗∗∗^	2.95	1.17
6. Negative Prototype Evaluation	0.02	–0.12^∗∗∗^	0.10^∗∗∗^	0.11^∗∗∗^	0.01	–	–0.23^∗∗^	4.74	1.17
7. Descriptive Norms	0.09^∗∗∗^	0.13^∗∗∗^	0.15^∗∗∗^	0.05^∗^	0.05^∗^	0.00	–	–0.16	0.62
*M*	16.35	–	3.10	2.35	2.70	3.36	0.03		
*SD*	1.45	–	1.40	0.57	1.07	1.27	0.68		

*Values above the diagonal present scores for Greece and below the diagonal present scores for Italy; ^∗^*p* < 0.05; ^∗∗^*p* < 0.005; ^∗∗∗^*p* < 0.001.*

### Multivariate Associations Between Moral Disengagement, PWM Constructs and Willingness to Engage in Cyberbullying

Two bootstrapped (5000 resamples) with bias corrected and accelerated (BCa) Confidence Intervals hierarchical regression models were used to assess the associations between moral disengagement, PWM constructs (risk prototype favorability), descriptive norms and willingness to engage in cyberbullying in the Greek and Italian samples, respectively. The models were completed in two steps, with the first step including basic demographic characteristics (age and gender), and moral disengagement, and the second stage including PWM constructs, namely positive and negative risk prototype favorability, and descriptive norms.

The first model (Greek sample) predicted 26.5% (Adjusted *R*^2^, *F* = 19.16, *p* < 0.001, multivariate *f*^2^ = 0.36) of the variance in willingness to engage in cyberbullying perpetration, and tolerance levels were high (>0.784) indicating no multicollinearity among predictor variables. In the first step of the analysis being male (β = −0.164, *p* = 0.002) and higher moral disengagement (β = 0.396, *p* < 0.001) were significantly associated with cyberbullying willingness. The addition of descriptive norms and risk prototype favorability in the second step of the analysis significantly increased the predicted variance of the model by 8.4% (*F*_change_ = 11.45, *p* < 0.001) but only descriptive norms, gender and moral disengagement were significantly associated with willingness to engage in cyberbullying. The results are summarized in Table 2.

**TABLE 2:** Psychological correlates of willingness to engage in cyberbullying in Greece and Italy.

		**Greece**				**Italy**			
			
		**B**	**β**	**95% CI for B**	**Adjusted *R*^2^**	**B**	**β**	**95% CI for B**	**Adjusted *R*^2^**
Step 1	Age	0.099	0.077	−0.035, 0.234	18.8%	0.016	0.017	−0.028, 0.061	13.1%
	Gender	–0.524	–0.164^∗∗^	−0.855, −0.192		–0.101	–0.036	−0.234, 0.033	
	Moral disengagement	2.442	–0.396^∗∗∗^	1.800, 3.084		0.868	0.355^∗∗∗^	0.752, 0.984	
Step 2	Age	0.094	0.073	−0.035, 0.224	26.5%	0.006	0.006	−0.038, 0.050	16.2%
	Gender	–0.444	−0.139^∗^	−0.771, −0.118		–0.138	−0.049^∗^	−0.271, −0.004	
	Moral disengagement	2.002	–0.324^∗∗∗^	1.354, 2.650		0.774	0.316^∗∗∗^	0.656, 0.891	
	Positive Prototype Evaluation	0.064	0.046	−0.084, 0.213		0.124	0.095^∗∗∗^	0.063, 0.184	
	Negative Prototype Evaluation	–0.004	–0.003	−0.158, 0.150		0.066	0.060^∗^	0.016, 0.116	
	Descriptive Norms	0.742	0.285^∗∗∗^	0.478, 1.007		0.284	0.140^∗∗∗^	0.191, 0.378	

*^∗^*p* < 0.05; ^∗∗^*p* < 0.005; ^∗∗∗^*p* < 0.001.*

The second model (Italian sample) predicted 16.2% (Adjusted *R*^2^, *F* = 52.25, *p* < 0.001, multivariate *f*^2^ = 0.19) of the variance in willingness to engage in cyberbullying, and tolerance levels were high (>0.879) indicating no multicollinearity among predictor variables. In the first step of the analysis moral disengagement (β = 0.355, *p* < 0.001) was significantly associated with willingness. Adding PWM constructs in the second step of the analysis significantly increased predicted variance by 3.2% (*F*_change_ = 20.57, *p* < 0.001) and the significant predictors of willingness to engage in cyberbullying included higher moral disengagement, being self-identified as male, positive and negative risk prototype favorability, and descriptive social norms. The results are summarized in Table 2.

### Indirect Effects of Moral Disengagement on Willingness to Engage in Cyberbullying

**FIGURE 1:**
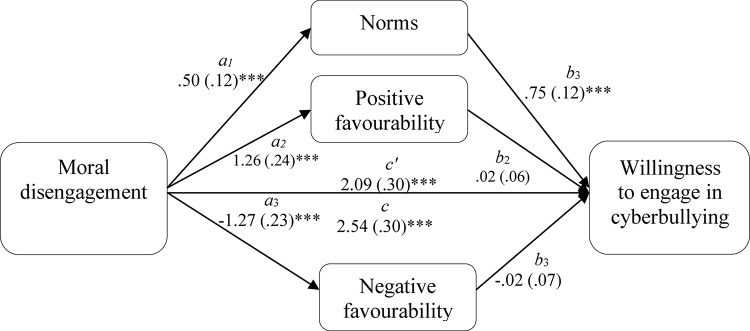
Total and indirect effect of moral disengagement on willingness to engage in cyberbullying in the Greek sample. The total (c) and the indirect effect (c’) of moral disengagement on willingness to engage in cyberbullying for the Greek sample are shown; unstandardized path coefficients are presented, with standard errors in brackets; ^∗^*p* < 0.05; ^∗∗^*p* < 0.005; ^∗∗∗^*p* < 0.001.

**FIGURE 2:**
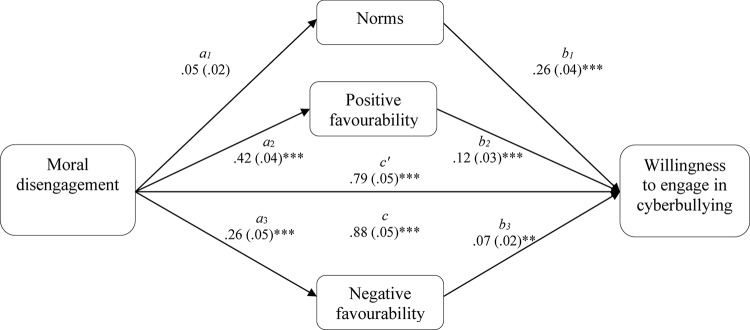
Total and indirect effect of moral disengagement on willingness to engage in cyberbullying in the Italian sample. The total (c) and the indirect effect (c’) of moral disengagement on willingness to engage in cyberbullying for the Italian sample are shown; unstandardized path coefficients are presented, with standard errors in brackets; ^∗^*p* < 0.05; ^∗∗^*p* < 0.005; ^∗∗∗^*p* < 0.001.

Multiple mediation modeling was used to assess the mediating role of risk prototype favorability and descriptive norms on the association between moral disengagement and willingness to engage in cyberbullying. For the analysis we used the SPSS Macro INDIRECT (Preacher and Hayes, 2008) with 5000 resamples and 95% confidence intervals, and the Sobel test (*z*) was used to enable effect size comparisons between the mediators. The results are summarized in Figures 1, 2. The analysis for the Greek sample showed that descriptive social norms significantly mediated the association between moral disengagement and willingness to engage in cyberbullying (*z* = 3.29, *p* = 0.001; Figure 1). The analysis for the Italian data showed that the association between moral disengagement and willingness to engage in cyberbullying was mediated by positive (*z* = 3.85, *p* < 0.001) and negative risk prototype favorability (*z* = 2.45, *p* < 0.05; Figure 2), but not descriptive social norms. A comparison of the mediation effects showed that positive risk prototype favorability had a significantly stronger (*p* < 0.05) mediation effect than negative ones.

## Discussion

In the present study we empirically examined a model of adolescents’ willingness to engage in cyberbullying that incorporated measures from Social Cognitive Theory (Bandura, 1999) and the PWM (Gibbons et al., 1998). The model was an extension of previous research on cyberbullying (Lazuras et al., 2013), and it was hypothesized that moral disengagement will be positively associated with willingness to engage in cyberbullying (Hypothesis 1), and that this association would be partly explained (mediated) by PWM constructs, namely descriptive social norms and risk prototype favorability (Hypothesis 2). The results supported the first hypothesis of the study by showing that moral disengagement was positively associated with willingness to engage in cyberbullying in both countries. Instead of TPB-based intention measures, in the present study we employed a more situation-based behavioral willingness measure to reflect the behavioral tendency to engage in cyberbullying perpetration in specific situations and social contexts in the future (Gibbons et al., 1998; Todd et al., 2016). This is in line with previous research showing a positive correlation between higher levels of moral disengagement and the tendency/intention to engage in cyberbullying perpetration (e.g., Lazuras et al., 2013; Kowalski et al., 2014; Bussey et al., 2015). Additionally, our results further extend the findings by Lazuras et al. (2013) by indicating that behavioral willingness can provide a useful alternative to TPB-based intention measures when assessing the association between moral disengagement and adolescents’ tendencies to engage in cyberbullying perpetration.

The present findings only partially supported the second hypothesis of the study. In particular, different variables emerged as mediators in the two countries. More specifically, in the Italian sample, moral disengagement retained a significant effect on willingness after PWM constructs were controlled for and this is in line with previous research (e.g., Lazuras et al., 2013). Multiple mediation modeling further showed that the association between moral disengagement and willingness was partly explained by risk prototype favorability evaluations – with positive evaluations exhibiting a stronger mediation effect than negative ones. In contrast descriptive social norms did not significantly mediated the moral disengagement-willingness relationship in this sample. In contrast, in the Greek sample, the association between moral disengagement and willingness to engage in cyberbullying was partly explained only by descriptive social norms. On the other hand, risk prototype favorability evaluations did not have a significant mediation effect. Those differences in mediation effects could be attributed to country-specific variation. That is, although moral disengagement was invariantly associated with willingness to engage in cyberbullying in both countries when the prototype favorability evaluations and descriptive social norms were accounted for in the model, still the mediating variables differed with prototype favorability evaluations being more relevant for the Italian participants, and descriptive social norms being more relevant for the Greek participants. Future research is needed to further confirm these associations, but we can tentatively explain our results in the following ways. In the Italian sample, risk prototype evaluation of the “typical” cyberbullying perpetrators serve as potential risk factors for the tendency to engage in cyberbullying under different situations. In the context of social cognitive theory this may mean that such evaluations facilitate the moral disengagement process (e.g., if the typical person who engages in cyberbullying is cool then it is OK to engage in cyberbullying). On the other hand, in the Greek sample the effect of moral disengagement on cyberbullying willingness seems to be facilitated by a more automatic normative process (e.g., if most people like me are doing it, then it cannot be that bad) that relies on the *perceived* sheer number of referent others who engage in cyberbullying than on the evaluation of the perpetrator’s attributes as positive or negative. In this respect, descriptive social norms facilitate moral disengagement processes, and this is in line with the proposition that normative processes allow people to make an “agentic shift” in justifying their actions – a mechanism which may further enable diffusion of responsibility (Osofsky et al., 2005; Bandura, 2016). In other words, morally disengaging from cyberbullying can be facilitated when people find the normative excuses to justify their behavior. Of course, these assumptions require further empirical examination with prospective designs that will enable us to draw more robust conclusions about the temporal associations between the constructs under study.

Overall, the findings of the present study indicated that although moral disengagement is a strong predictor of willingness to cyberbully, its effect can be mediated by different constructs under different circumstances. Recently, Travlos et al. (2018) demonstrated that different personal (i.e., gender and age) variables may influence the effect of moral disengagement on traditional bullying behavior. The present study including participants of different ethnic background, age and gender distribution, also suggested that such personal and cultural variables may be responsible for the differential processes found in predicting the decision to cyberbully. Future studies should take into account such variables when attempting to investigate the decision making process toward cyberbullying too.

The study is not free of limitations. Firstly, it is a cross-sectional study and causal inferences cannot be made, since the data describe the association among the variables under study. Secondly, the present study is based on self-reports and it possible that some responses were influenced by social desirability, even if the anonymity was ensured. Further studies with a longitudinal design and including different tools for data collections will be very useful to confirm our findings. Furthermore, the two samples used in the present study are not fully comparable in terms of size and mean age. These differences, especially the small sample in Greece, did not allow for the use of more sophisticated analyses (i.e., SEM or path analysis, or multilevel analysis) that would provide a more comprehensive understanding of multilevel effects on the behavior and the related psychological processes we studied (e.g., distinguish between school-level and student-level influences on moral disengagement or self-reported cyberbullying behavior). In addition, the reliability of the willingness toward cyberbullying and prototype perceptions were marginally acceptable and caution is needed in interpreting the findings with respect to this variable. Notwithstanding these limitations, this is among the first studies to investigate the joint effect of moral disengagement and prototype perceptions on willingness to cyber bully. Importantly, in the present study data from two countries are presented and demonstrate that moral disengagement is consistently a strong predictor of transgressive behaviors, such as cyberbullying. Overall, the present study provides valuable information on the precursors of cyberbullying behavior in adolescence and can inform future research on the psychological mechanisms underpinning cyberbullying behavior.

## Ethics Statement

The study design has been approved by the Hellenic Ministry of Education and Religious Affairs and is in line with the Code of Ethics in Research of the Arsitotle University of Thessaloniki.

## Author Contributions

All authors contributed equally in the planning of the study, data collection, and manuscript preparation.

## Conflict of Interest Statement

The authors declare that the research was conducted in the absence of any commercial or financial relationships that could be construed as a potential conflict of interest.

## Footnotes

1 See for example http://appsso.eurostat.ec.europa.eu/nui/show.do?dataset=ilc_lvps01&lang=en
